# Determinants of hospitalization expenditure for pediatric congenital heart disease: a single-center study from Xinjiang, China

**DOI:** 10.3389/fpubh.2025.1714957

**Published:** 2026-01-12

**Authors:** Lizha Jiangabieke, Azhamati Azhati, Huiling Xie

**Affiliations:** 1 Department of Health Economics, School of Public Health, Xinjiang Medical University, Urumqi, China; 2 Department of Pediatric Cardiothoracic Surgery, The First Affiliated Hospital of Xinjiang Medical University, Urumqi, China

**Keywords:** path analysis, children, China, congenital heart disease, determinants, hospitalization costs, mediation analysis

## Abstract

**Background:**

This study aimed to identify key determinants of hospitalization costs for children aged 0–14 years with congenital heart disease (CHD) at a tertiary hospital in Xinjiang, China, to provide evidence for reducing the financial burden on families and improving health insurance policies.

**Methods:**

We conducted a retrospective analysis of medical records from 2,811 pediatric patients who underwent CHD surgery between September 2013 and September 2024. Potential influencing factors were screened using non-parametric tests, and mediating effects were examined through the Bias-Corrected Bootstrap method with 5,000 repetitions. Path analysis modeling was performed with AMOS 24.0 to clarify direct and indirect pathways among variables affecting hospitalization costs.

**Results:**

Median hospitalization costs for CHD patients showed an initial increase followed by a progressive decline, with sustained reduction after 2019. Costs for open-heart surgery consistently exceeded those for interventional procedures, with the former dominated by treatment fees and the latter primarily composed of material costs. Several factors were significantly associated with higher costs, including age under 2 years, absence of health insurance, complex CHD, open-heart surgery, and hospital stays lasting 20 days or longer (all *P* < 0.05). Path analysis indicated that older age (β = −0.047) indirectly reduced costs through the “treatment approach → length of stay” pathway. In contrast, both disease type (β = 0.087) and severity (β = 0.061) demonstrated positive indirect effects along the same pathway. Health insurance (β = −0.045) contributed to cost reduction indirectly through its association with shorter hospitalization.

**Conclusion:**

Multiple clinical and socioeconomic factors influence hospitalization costs for children with CHD. To minimize the risk of catastrophic health expenditures for families, we recommend prioritizing interventional procedures, optimizing insurance reimbursement strategies, and enhancing awareness and enrollment in neonatal health insurance programs.

## 1 Introduction

Congenital heart disease (CHD) refers to a group of structurally heterogeneous cardiovascular malformations caused by abnormal development of the heart and major vessels during fetal growth (1). As the most common category of birth defects, CHD has a reported incidence of 7–9 per 1,000 live births (2). Global epidemiological studies estimated a CHD prevalence of approximately 1.8 per 100 live births in 2017 (3). According to the Global Burden of Disease Study, approximately 3 million newborns were diagnosed with CHD in 2019, while 13.3 million individuals worldwide were living with various forms of congenital cardiac abnormalities (4). In China, the documented incidence of CHD has increased substantially, rising 4.3-fold from 3.3 per 1,000 perinatal infants (from 28 weeks of gestation to 7 days after birth) in 2010 to 17.3 per 1,000 in 2020 (5). Accounting for nearly one-third of all birth defects, CHD represents a significant global public health challenge.

Both open-heart surgery and interventional procedures serve as fundamental treatment approaches for CHD (6), with contemporary techniques enabling over 90% of affected children to reach adulthood (7–9). Nevertheless, the associated treatment expenses place a considerable economic burden on families and healthcare systems (10–12), a challenge particularly pronounced in low- and middle-income regions with constrained medical resources (3). Recent data from Xinjiang (2024) revealed a median financial burden of ¥44,140 (approximately US$6,217) for families of children with CHD, with hospitalization expenses comprising 86% of this total (13). Therefore, detailed analysis of hospitalization costs is essential for developing targeted strategies to alleviate household financial stress, improve hospital cost containment, foster equitable resource distribution, and support evidence-based health policy formulation.

Path analysis provides an advanced methodological framework for healthcare cost research by elucidating both direct and indirect relationships between variables, offering deeper insights than conventional regression approaches (14, 15). Most existing investigations into CHD hospitalization costs have primarily examined adult populations or utilized multi-center datasets, predominantly employing traditional regression techniques that may not fully capture the complex intermediary pathways among determinants. Research specifically addressing pediatric CHD in Xinjiang remains limited, particularly studies that integrate path analysis with the ongoing Diagnostic-Related Group (DRG) and Disease Intervention Program (DIP) health insurance status payment reforms. To address these gaps, this study employs path analysis of Hospital Information System (HIS) data from a major tertiary hospital in Xinjiang to systematically elucidate the mechanisms driving hospitalization costs for pediatric CHD patients in the region. The findings aim to provide an empirical foundation for reducing family financial stress, enhancing the efficiency of health insurance status fund allocation, and supporting evidence-based health policy development.

## 2 Methodology

### 2.1 Data sources

This study utilized data obtained from a large tertiary general hospital in Ürümqi, Xinjiang. The institution's well-established pediatric cardiothoracic surgery department manages a substantial patient volume with broad geographical coverage, thereby ensuring both clinical representativeness and high data quality for the analyzed cases.

Congenital heart disease (CHD) cases were classified into simple or complex types according to the International Classification of Diseases (ICD) guidelines and established clinical criteria (16). Simple CHD included nine specific conditions such as ventricular septal defect, atrial septal defect, and patent ductus arteriosus. Complex CHD comprised eight conditions including tetralogy of Fallot, transposition of the great arteries, and single ventricle heart. Based on this classification scheme, complex CHD was categorized as ‘severe' while simple CHD was designated as “non-severe”. This clinical severity classification was determined by attending physicians according to the final diagnosis documented on the front page of each medical record.

The dataset included comprehensive information on sociodemographic characteristics (including age, gender, and health insurance status type), clinical features (such as disease type, severity classification, treatment approach, duration of hospitalization, and surgical year), and detailed hospitalization cost components.

### 2.2 Composition of hospitalization costs

Hospitalization costs were defined according to the Hospital Information System (HIS) classification framework, comprising eight distinct categories: comprehensive medical fees, nursing fees, diagnostic fees, treatment fees, medication fees, laboratory fees (covering costs for blood, urine, and other monitoring tests), material fees, and other miscellaneous fees. Specifically, comprehensive medical fees included charges for general medical services and basic therapeutic procedures. Diagnostic fees encompassed pathological examinations, laboratory diagnostics, medical imaging, and clinical diagnostic procedures. Treatment fees consisted of non-surgical therapeutic interventions, surgical procedures, and traditional Chinese medicine treatments. Medication fees covered Western pharmaceuticals, Chinese patent medicines, and herbal preparations. Material fees included disposable medical supplies for examination, treatment, and surgical purposes. The “other fees” category incorporated all remaining expenses not classified elsewhere.

### 2.3 Data preprocessing

Following data extraction from the Hospital Information System (HIS), a comprehensive data cleaning process was implemented across all variables. Critical variables—including hospitalization costs, treatment approaches, and disease diagnoses—contained missing values in < 1% of cases. These incomplete records were excluded from subsequent analysis using listwise deletion. To account for inflation and enable meaningful temporal comparison, all cost data from 2013 to 2024 were standardized to 2024 values using a 5% annual discount rate. The study employed a retrospective design with predefined inclusion and exclusion criteria. Eligible participants included: (1) domestic pediatric patients aged ≤ 14 years with a confirmed CHD diagnosis; and (2) those who underwent either surgical or interventional procedures at the study institution. Exclusion criteria comprised: (1) CHD patients hospitalized without receiving surgical or interventional treatment; and (2) patients presenting with concurrent comorbid conditions. After applying these screening procedures, a final cohort of 2,811 pediatric patients with complete datasets was established for all subsequent analyses. The study utilized exclusively anonymized medical record data without any personal identifiers, thereby qualifying for exemption from ethical approval requirements according to institutional guidelines.

### 2.4 Statistical analysis

Data management was performed using Microsoft Excel, while all statistical analyses were conducted with SPSS 27.0 and AMOS 24.0. The analyzed variables encompassed gender, age, health insurance status, disease type, disease severity, treatment approach, length of hospitalization, surgical year, and hospitalization costs.

The Shapiro–Wilk test confirmed a non-normal distribution for hospitalization costs (*P* < 0.001). Accordingly, categorical variables are presented as frequencies with percentages, and continuous variables as medians with interquartile ranges. Group comparisons were performed using the Mann–Whitney *U* test for two groups and the Kruskal–Wallis *H* test for multiple groups. Variables demonstrating significant associations (*P* < 0.05) in univariate analysis were included in the subsequent path analysis model. To meet the normality assumptions of path analysis, logarithmic transformation was applied to skewed variables including hospitalization costs.

Mediation analyses examining the roles of “treatment approach” and “length of hospital stay” were conducted using the Bias-Corrected Bootstrap method with 5,000 resamples. Statistical significance for mediation effects was determined by 95% confidence intervals excluding zero. Path analysis models were developed using AMOS 24.0 to identify both direct and indirect pathways through which each variable influences hospitalization costs.

## 3 Results

### 3.1 Hospitalization costs for pediatric congenital heart disease patients (2013–2024)

#### 3.1.1 Trends in total hospitalization costs

Between 2013 and 2024, median hospitalization costs for pediatric CHD patients demonstrated a distinct “rise-then-fall” pattern. After increasing from 2013 levels and reaching a peak of ¥64,048.65 in 2015, costs fluctuated before declining to ¥52,044.06 by 2019. This downward trend continued persistently after 2019, with costs falling to their lowest observed value of ¥29,690.08 in 2024 (Table 1).

**Table 1:** Median hospitalization costs of children with CHD from 2013 to 2024.

**Year/years**	**Number/ Example**	**Comprehensive medical costs**	**Care costs**	**Diagnosis fee**	**Medical expense**	**Medications**	**Laboratory fees**	**Material cost**	**Miscellaneous expenses**	**Median cost of hospitalization**
2013	56	6,184.37	1,171.58	9,757.91	12,544.48	13,085.17	1,087.78	12,251.20	170.32	56,247.56
2014	222	6,614.85	1,115.47	9,833.23	13,093.08	11,272.69	1,266.47	13,579.20	156.05	57,169.94
2015	257	6,216.95	1,006.81	9,995.21	13,937.13	12,072.30	1,326.39	17,033.20	139.31	64,048.65
2016	292	4,432.74	701.79	8,247.90	10,538.69	11,855.15	709.18	19,700.77	114.95	61,666.91
2017	296	5,125.01	831.60	7,046.06	10,881.46	8,791.37	757.02	15,406.47	81.33	53,966.39
2018	271	6,360.80	998.51	8,470.25	12,576.62	10,204.59	940.75	16,496.47	93.92	58,014.01
2019	323	7,644.93	1,176.73	8,547.26	11,866.87	5,834.76	0.00	20,919.58	15.32	52,044.06
2020	205	6,322.46	903.12	8,767.45	11,241.00	6,144.80	164.09	12,534.87	0.00	48,341.00
2021	308	5,469.20	493.73	7,356.42	8,845.41	5,245.89	0.00	12,095.73	0.00	43,483.34
2022	186	3,352.29	646.62	6,941.34	7,633.71	2,796.97	0.00	15,889.55	0.00	36,010.58
2023	249	4,770.15	787.50	7,390.95	9,303.00	2,586.62	110.25	10,124.51	0.00	35,381.65
2024	146	3,917.50	952.50	6,002.50	8,041.50	1,746.63	0.00	8,698.56	0.00	29,690.08

Unit: Yuan.

#### 3.1.2 Comparative analysis of costs and composition: open-heart vs. interventional procedures

Throughout the 2013–2024 study period, hospitalization costs for open-heart surgical procedures remained consistently higher than those for interventional approaches. Costs for open-heart surgery demonstrated fluctuations before 2020, reaching ¥55,624.93 in that year before entering a steady decline to ¥33,937.77 by 2024. Meanwhile, interventional procedure costs showed an overall downward trajectory after peaking at ¥62,736.26 in 2015, ultimately decreasing to ¥22,105.25 in 2024, with the most substantial reductions occurring in recent years.

Analysis of cost structures revealed distinct patterns between the two approaches. Treatment fees represented the predominant cost component in open-heart surgeries, increasing from 22.30% of total costs in 2013 to 32.20% in 2024. In contrast, material costs constituted the predominant component for interventional procedures, accounting for the highest proportion of total costs since 2014, albeit with a fluctuating downward trend. with proportions ranging from 29.26% to 54.55% across the study period (see Tables 2–5 for detailed breakdowns).

**Table 2:** Median cost of open-heart surgery hospitalization for children with CHD from 2013 to 2024.

**Year/years**	**Comprehensive medical costs**	**Care costs**	**Diagnosis fee**	**Medical expense**	**Medications**	**Laboratory fees**	**Material cost**	**Miscellaneous expenses**	**Median cost of hospitalization**
2013	6,184.37	1,171.58	9,757.91	12,544.48	13,085.17	1,087.78	12,251.20	170.32	56,247.56
2014	6,614.85	1,115.47	9,833.23	13,093.08	11,272.69	1,266.47	13,579.20	156.05	56,703.10
2015	6,846.40	1,141.00	10,383.04	14,693.02	12,748.28	1,428.00	16,609.72	151.72	64,449.27
2016	5,616.55	809.65	8,892.80	12,959.50	13,020.00	967.73	17,364.08	132.68	59,148.20
2017	6,586.29	1,035.63	8,475.32	12,671.29	9,804.41	987.78	12,258.86	99.62	55,440.99
2018	6,360.80	998.51	8,470.25	12,576.62	10,204.59	940.75	16,496.47	93.92	58,014.01
2019	8,316.89	1,452.41	9,319.41	14,845.71	7,558.48	446.70	9,282.17	15.32	53,756.94
2020	9,867.48	1,493.25	10,457.00	14,412.26	7,519.02	401.12	10,727.27	0.00	55,624.93
2021	7,782.42	677.21	8,006.42	13,206.19	7,176.77	329.34	9,442.98	0.00	45,292.99
2022	6,589.92	1,592.01	7,971.08	12,837.51	4,999.46	347.29	8,893.33	11.03	42,453.63
2023	6,711.86	1,659.00	8,756.48	12,251.93	4,013.54	341.25	8,338.53	10.50	41,334.44
2024	4,902.00	1,694.00	6,832.00	10,929.00	2,560.41	325.00	7,870.36	0.00	33,937.77

Unit: Yuan.

**Table 3:** Cost composition of open-heart surgery hospitalization for children with CHD from 2013 to 2024.

**Year/years**	**Comprehensive medical costs**	**Care costs**	**Diagnosis fee**	**Medical expense**	**Medications**	**laboratory fees**	**Material cost**	**Miscellaneous expenses**
2013	10.99	2.08	17.35	22.30	23.26	1.93	21.78	0.30
2014	11.67	1.97	17.34	23.09	19.88	2.23	23.95	0.28
2015	10.62	1.77	16.11	22.80	19.78	2.22	25.77	0.24
2016	9.50	1.37	15.03	21.91	22.01	1.64	29.36	0.22
2017	11.88	1.87	15.29	22.86	17.68	1.78	22.11	0.18
2018	10.96	1.72	14.60	21.68	17.59	1.62	28.44	0.16
2019	15.47	2.70	17.34	27.62	14.06	0.83	17.27	0.03
2020	17.74	2.68	18.80	25.91	13.52	0.72	19.29	0.00
2021	17.18	1.50	17.68	29.16	15.85	0.73	20.85	0.00
2022	15.52	3.75	18.78	30.24	11.78	0.82	20.95	0.03
2023	16.24	4.01	21.18	29.64	9.71	0.83	20.17	0.03
2024	14.44	4.99	20.13	32.20	7.54	0.96	23.19	0.00

Unit: %.

**Table 4:** Median hospitalization cost of interventional surgery for CHD children from 2013 to 2024.

**Year/Year**	**Comprehensive medical costs**	**Care costs**	**Diagnosis fee**	**Medical expense**	**Medications**	**Laboratory fees**	**Material cost**	**Miscellaneous expenses**	**Median cost of hospitalization**
2013	0.00	0.00	0.00	0.00	0.00	0.00	0.00	0.00	0.00
2014	1,600.80	341.25	7,594.72	11,539.90	2,177.29	0.00	27,889.67	136.50	51,280.14
2015	3,148.42	508.84	7,291.24	7,426.21	10,535.64	0.00	34,515.53	102.08	63,273.62
2016	3,314.67	518.59	7,632.53	7,184.13	10,296.76	0.00	33,810.12	103.13	62,280.26
2017	3,507.73	645.16	6,156.06	9,009.66	7,692.45	0.00	26,008.77	70.07	52,732.98
2018	7,002.00	1098.95	7,577.24	11,849.19	8,798.67	0.00	15,854.34	58.82	54,192.39
2019	6,225.22	950.19	8,000.37	8,611.07	4,450.23	0.00	22,366.36	15.32	50,868.96
2020	3,332.01	469.19	7,266.30	7,883.77	3,609.43	0.00	21,127.36	0.00	44,033.55
2021	3,316.16	347.29	6,477.49	7,357.86	3,018.54	0.00	20,727.73	0.00	41,410.49
2022	1,847.79	201.76	5,909.40	6,687.77	1,699.48	0.00	18,860.61	0.00	34,423.81
2023	1,505.18	160.65	5,691.00	6,240.15	868.44	0.00	14,238.37	0.00	28,726.60
2024	1,377.00	263.00	4,817.00	5,957.50	619.10	0.00	8,764.98	0.00	22,105.25

Unit: Yuan.

**Table 5:** Analysis of the composition of hospitalization costs for interventional surgery in children with CHD from 2013 to 2024.

**Year/years**	**Comprehensive medical costs**	**Care costs**	**Diagnosis fee**	**Medical expense**	**Medications**	**Laboratory fees**	**Material cost**	**Miscellaneous expenses**
2013	–	–	–	–	–	–	–	–
2014	3.12	0.67	14.81	22.50	4.25	0.00	54.39	0.27
2015	4.98	0.80	11.52	11.74	16.65	0.00	54.55	0.16
2016	5.32	0.83	12.26	11.54	16.53	0.00	54.29	0.17
2017	6.65	1.22	11.67	17.09	14.59	0.00	49.32	0.13
2018	12.92	2.03	13.98	21.87	16.24	0.00	29.26	0.11
2019	12.24	1.87	15.73	16.93	8.75	0.00	43.97	0.03
2020	7.57	1.07	16.50	17.90	8.20	0.00	47.98	0.00
2021	8.01	0.84	15.64	17.77	7.29	0.00	50.05	0.00
2022	5.37	0.59	17.17	19.43	4.94	0.00	54.79	0.00
2023	5.24	0.56	19.81	21.72	3.02	0.00	49.57	0.00
2024	6.23	1.19	21.79	26.95	2.80	0.00	39.65	0.00

Unit: %.

To directly compare resource use between the two treatment approaches during the study period, Table 6 presents the median length of stay and total hospitalization costs. Children receiving open-heart surgery had a longer median hospitalization (14.00 days) and higher total costs (¥54,573.63) than those treated with interventional surgery (9.00 days, ¥47,233.91).

**Table 6:** Comparison of hospitalization duration and costs between open-heart surgery and interventional surgery groups.

**Variable**	**Open-heart surgery (*n* = 1,809)**	**Interventional surgery (*n* = 1,002)**
Length of stay (days), Median (Q1, Q3)	14.00 (9.50, 18.50)	9.00 (7.00, 11.00)
Total hospitalization costs (¥), Median (Q1, Q3)	54,573.63 (45,787.63, 63,359.63)	47,233.91 (42,829.91, 51,637.91)

### 3.2 Demographic characteristics and univariate analysis

The study cohort comprised 2,811 pediatric CHD patients, with female patients accounting for 53.98%. The largest age group was 2–5 years (36.04%), and a notable 52.80% of patients lacked health insurance status coverage. Atrial septal defect (44.47%) and ventricular septal defect (32.19%) represented the most common diagnoses, while 54.54% of cases were classified as severe CHD. Open-heart procedures constituted 64.35% of all interventions, and the majority of hospitalizations (61.47%) lasted between 7 and 15 days. The year 2019 accounted for the highest treatment volume (11.49%).

Univariate analysis demonstrated significant associations between hospitalization costs and all examined variables: gender, age, insurance status, disease type, disease severity, treatment approach, hospitalization duration, and surgical year (all *P* < 0.05). Variable coding schemes are detailed in Table 7, with complete demographic and clinical characteristics presented in Table 8.

**Table 7:** Value assignments for variables in the analysis of hospitalization costs.

**Variable**	**Value assignment**
Gender	Male = 1, Female = 2
Age group	< 2=1,2~ < 5=2,5~ < 8=3,8~ < 12=4,12~ < 14=5
Health insurance status	Uninsured = 0, Insured = 1
Disease type	Atrial septal defect = 1, ventricular septal defect = 2, patent ductus arteriosus = 3, tetralogy of fallot = 4, atrioventricular septal defect = 5, partial anomalous pulmonary venous connection = 6, aortic stenosis = 7, total anomalous pulmonary venous connection = 8, pulmonary stenosis = 9, other = 10
Disease severity	Severe =1, Mild =2
Treatment modality	Open-heart surgery = 1, interventional procedure = 2
Length of hospital stay	< 7=1, 7~ < 11=2, 11~ < 15=3, 15~ < 20=4, ≥20=5
Treatment year	2013=1, 2014=2, 2015=3, 2016=4, 2017=5, 2018=6, 2019=7, 2020=8, 2021=9, 2022=10, 2023=11, 2024=12

Disease severity was classified as severe for complex CHD (e.g., tetralogy of Fallot, transposition of the great arteries) and non-severe for simple CHD (e.g., atrial septal defect, ventricular septal defect).

**Table 8:** Baseline characteristics and univariate analysis of hospitalization costs in pediatric CHD patients.

**Variable**	**Group**	**Frequency**	**Proportion (%)**	**Median hospitalization cost (CNY)**	***Z*/*H***	***P***
Gender	Male	1,296	46.10	52,400.46	−4.280	< 0.001
	Female	1,515	53.98	49,953.9		
Age group	< 2	561	19.96	61,657.91	288.495	< 0.001
	2 < 5	1,013	36.04	52,360.28		
	5 < 8	617	21.96	46,987.69		
	8 < 12	430	15.28	44,809.59		
	12 < 14	190	6.76	45,120.19		
Health insurance status	Uninsured	1,485	52.80	53,872.79	−7.988	< 0.001
	Insured	1,326	47.20	47,746.36		
Disease type	Atrial Septal Defect	1,250	44.47	46,921.87	988.289	< 0.001
	Ventricular Septal Defect	905	32.19	58,827.99		
	Patent Ductus Arteriosus	363	12.91	32,326.5		
	Tetralogy of Fallot	114	4.06	85,042.92		
	Atrioventricular Septal Defect	28	0.99	51,928.08		
	Partial Anomalous Pulmonary Venous Connection	25	0.90	51,717.68		
	Aortic Stenosis	18	0.64	58,207.31		
	Total Anomalous Pulmonary Venous Connection	16	0.57	91,398.6		
	Pulmonary Stenosis	11	0.39	64,231.09		
	Other	81	2.88	77,980.98		
Disease severity	Severe	1,533	54.54	52,468.64	−6.827	< 0.001
	Mild	1,278	45.46	50,121.31		
Treatment modality	Open-heart surgery	1,809	64.35	54,573.63	−10.884	< 0.001
	Interventional procedure	1,002	35.65	47,233.91		
Length of hospital stay	< 7	133	4.73	35,122.49	963.176	< 0.001
	7 < 11	903	32.12	44,589.92		
	11 < 15	825	29.35	50,309.46		
	15 < 20	451	16.05	58,355.13		
	≥20	499	17.75	82,874.86		
Treatment year	2013	56	1.99	56,247.56	732.390	< 0.001
	2014	222	7.89	56,627.47		
	2015	257	9.14	64,048.65		
	2016	292	10.37	61,666.91		
	2017	296	10.57	53,966.39		
	2018	271	9.64	52,463.44		
	2019	323	11.49	52,044.06		
	2020	205	7.29	48,341.00		
	2021	308	10.95	43,483.34		
	2022	186	6.62	36,010.58		
	2023	249	8.86	35,381.65		
	2024	146	5.19	29,690.08		

### 3.3 Path analysis of influencing factors

Univariate analysis demonstrated that gender, age, health insurance status, disease type, disease severity, treatment approach, hospitalization duration, and surgical year all demonstrated statistically significant associations with hospitalization costs (*P* < 0.05). To further investigate the magnitude and pathways of relationships among these influencing factors, path analysis was conducted. The structural equation model demonstrated acceptable fit, with the following indices exceeding the 0.8 threshold: GFI = 0.943, NFI = 0.837, IFI = 0.838, and CFI = 0.837. These results support the statistical appropriateness of the proposed model (Table 9).

**Table 9:** Goodness-of-fit indices for the structural equation model.

**Fit index**	**Recommended value**	**Model value**
Goodness-of-fit index (GFI)	>0.8	0.943
Normed fit index (NFI)	>0.8	0.837
Incremental fit index (IFI)	>0.8	0.838
Comparative fit index (CFI)	>0.8	0.837

Path analysis identified several variables with significant direct effects on hospitalization costs. Gender (β = −0.048), age (β = −0.083), health insurance status (β = −0.056), treatment approach (β = −0.120), and treatment year (β = −0.392) all demonstrated statistically significant negative relationships with costs (all *P* < 0.001). Conversely, disease type (β = 0.043, *P* = 0.003), disease severity (β = 0.178, *P* < 0.001), and length of hospital stay (β = 0.519, *P* < 0.001) showed significant positive associations with hospitalization costs (Table 10).

**Table 10:** Direct effects of independent variables on hospitalization costs.

**Pathway**	**Standardized estimate (β)**	**S.E**.	***P***
Hospitalization costs < – Gender	−0.048	0.005	< 0.001
Hospitalization costs < – Age group	−0.083	0.002	< 0.001
Hospitalization costs < – Health insurance status	−0.056	0.005	< 0.001
Hospitalization costs < – Disease type	0.043	0.001	0.003
Hospitalization costs < – Disease severity	0.178	0.005	< 0.001
Hospitalization costs < – Treatment modality	−0.120	0.004	< 0.001
Hospitalization costs < – Length of hospital stay	0.519	0.002	< 0.001
Hospitalization costs < – Treatment year	−0.392	0.001	< 0.001

#### 3.3.1 Mediation effects analysis

**Figure 1:**
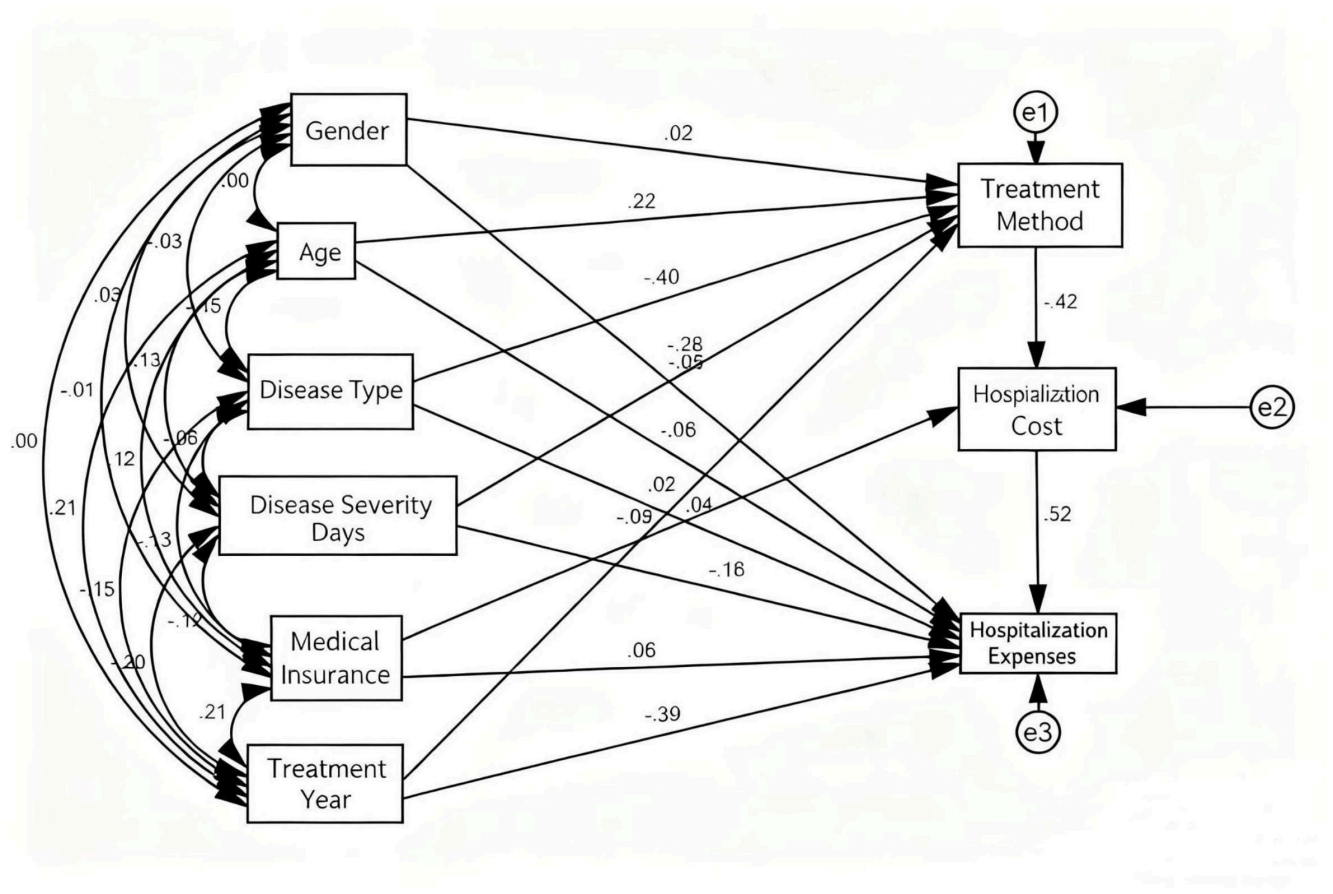
Path diagram illustrating the relationships between variables and hospitalization costs.

Several variables significantly influenced hospitalization costs through specific mediation pathways. Age, disease severity, disease type, and health insurance status demonstrated statistically significant indirect effects via the “treatment approach → length of stay” pathway, with mediation effect values of −0.047, 0.061, 0.087, and −0.045, respectively (detailed in Table 10). These findings indicate that older age and health insurance coverage were associated with cost reduction by increasing the likelihood of selecting interventional treatment and shortening hospitalization duration. Conversely, greater disease severity and complex disease types contributed to higher costs through increased utilization of open-heart surgery and prolonged hospital stays. The indirect effects of gender and treatment year were not statistically significant (Table 11). The comprehensive path analysis results are visually summarized in Figure 1.

**Table 11:** Indirect (mediating) effects on hospitalization costs.

**Mediating pathway**	***S.E*.**	**Standardized estimate (β)**	**95% CI**	***P***
Gender → Treatment Modality → Length of Stay → Costs	0.003	−0.004	−0.009 0.004	0.346
Age → Treatment Modality → Length of Stay → Costs	0.004	−0.047	−0.056 −0.039	0.004
Disease Type → Treatment Modality → Length of Stay → Costs	0.005	0.087	0.078 0.097	0.007
Disease Severity → Treatment Modality → Length of Stay → Costs	0.004	0.061	0.052 0.067	0.025
Treatment Year → Treatment Modality → Length of Stay → Costs	0.004	−0.003	−0.010 0.004	0.365
Health Insurance → Length of Stay → Costs	0.01	−0.045	−0.061 −0.025	0.032

## 4 Discussion

### 4.1 Analysis of hospitalization costs and their composition in pediatric CHD patients

#### 4.1.1 Analysis of total cost trends

Between 2013 and 2024, median hospitalization costs for pediatric CHD patients demonstrated a distinct initial-increase-then-decline pattern, with sustained reduction observed after 2019. This trajectory coincides with China's phased healthcare reform initiatives: the 2015 expansion of diagnosis-related group (DRG) payment pilots, the 2017 nationwide elimination of drug price markups, and the 2019 volume-based procurement policy for high-value medical consumables potentially contributed to moderated cost structures. Pharmaceutical expenses, no longer subject to markup premiums, decreased substantially from ¥13,085.17 in 2013 to ¥1,746.63 in 2024, representing an 86.7% reduction. Simultaneously, material costs—the largest expenditure category—declined from their 2019 peak of ¥20,919.58 to ¥8,698.56 in 2024.

A noticeable shift toward minimally invasive interventional procedures, which generally incur lower costs than open-heart surgeries, has emerged within treatment patterns. Concurrently, treatment costs for open-heart procedures were contained through technical standardization and optimized postoperative management, with overall treatment expenses decreasing from the 2015 peak of ¥13,937.13 to ¥8,041.50 in 2024, thereby reducing perioperative resource utilization. Nevertheless, other factors including medical technology advancements and streamlined clinical pathways may have collectively influenced these outcomes. This study reports observed associations rather than establishing definitive causal relationships between specific policies and cost variations.

However, when examining the financial burden on households, the median hospitalization cost for pediatric CHD patients during 2013–2024 reached ¥49,672.01, approximately 1.61 times the 2024 per capita disposable income in Xinjiang [¥30,899 (17)]. According to World Health Organization standards, out-of-pocket medical expenditures exceeding 10% of household disposable income constitute catastrophic health spending. With a mean medical cost reimbursement ratio of 54% in this study, the estimated median out-of-pocket expenditure was approximately ¥22,899, accounting for 74.1% of local per capita disposable income—substantially surpassing the catastrophic expenditure threshold. This substantial financial burden highlights critical gaps in the current health security system in mitigating direct household financial stress but also reveals fundamental equity gaps within the healthcare security system, indicating an urgent need for comprehensive policy reform.

To address these challenges, developing a multi-layered protection strategy aligned with Xinjiang's economic development level and CHD treatment cost structure becomes imperative. Health insurance authorities should consider substantially increasing the overall reimbursement rate for CHD while implementing separate reimbursement policies for high-value consumables (such as interventional occluders) and complex surgical items. Furthermore, establishing an income- and severity-graded medical subsidy system would provide secondary financial assistance to families of severely ill children after basic insurance reimbursement. Simultaneously, standardizing clinical pathways and optimizing the complete care continuum from preoperative evaluation to postoperative follow-up would maintain treatment quality while containing non-essential expenditures. The coordinated implementation of these multidimensional policies represents a crucial approach to meaningfully reduce household financial burdens, prevent medical impoverishment, and advance the local healthcare security system toward greater equity and sustainability.

#### 4.1.2 Comparative analysis of costs between open-heart and interventional procedures

The principal treatment approaches for CHD include open-heart surgery and interventional procedures. During the 2013–2024 period, median hospitalization costs for open-heart surgery (¥54,593.02) consistently surpassed those for interventional approaches (¥47,215.90) in Xinjiang's pediatric population, although both modalities demonstrated overall declining trends. Specifically, open-heart surgery costs experienced a temporary increase to ¥55,624.93 in 2020 before commencing a steady decline to ¥33,937.77 by 2024. Meanwhile, interventional procedure costs displayed more substantial reduction following their 2015 peak (¥62,736.26), decreasing to ¥22,105.25 by 2024. These cost differentials primarily reflect inherent differences in resource utilization between surgical approaches, combined with the influential role of healthcare reform policies. The findings offer valuable empirical evidence for assessing both clinical value and cost-effectiveness of CHD treatment strategies within the regional context.

The elevated costs associated with open-heart surgery primarily stem from the requirement for cardiopulmonary bypass and frequent postoperative intensive care unit (ICU) admission to manage potential complications and infections, consequently consuming substantial medical resources (18). The composition of open-heart surgery costs has undergone notable transformation following healthcare reform initiatives. During 2013–2018, medication, materials, and treatment fees represented balanced expenditure categories. From 2019 onward, however, treatment fees emerged as the dominant cost component (comprising 27.62% in 2019 vs. 14.06% for medication and 17.27% for materials, Table 3), maintaining this leading position through 2024 (32.20%). This structural shift aligns with the policy orientation emphasized in the 2017 “Guiding Opinions on Establishing a Modern Hospital Management System,” which advocated for better recognition of healthcare professionals' labor value. Although the policy didn't produce a unidirectional increase in treatment fees, it established institutional support for their appropriate recalibration. The pre-implementation period (2013–2016) showed treatment fees averaging 22.25% of total costs, while post-implementation (2017–2024) saw this proportion rise to 28.34%. The notable increase in treatment fee proportion in 2019 was partially attributable to a “denominator effect” created by volume-based procurement of high-value consumables reducing material cost percentages. Although the treatment fee proportion subsequently declined to 29.16% in 2021, mainly due to optimized postoperative processes reducing other expenses, its absolute value (¥13,206.19) remained elevated compared to 2017 levels (¥12,671.29), confirming preserved valuation of technical labor services.

Post-2019, median treatment costs for open-heart surgery demonstrated a notable decline (Table 2: decreasing from ¥14,845.71 in 2019 to ¥10,929.00 in 2024). This reduction coincided with cost-containment initiatives including Diagnosis-Related Groups (DRG) pilot programs and standardized clinical pathway implementation. Healthcare institutions maintained the value of clinical labor while controlling treatment expenditures through optimized perioperative management protocols. Simultaneously, comprehensive medical fees declined from ¥8,316.89 in 2019 to ¥4,902.00 in 2024 (Table 2), suggesting potential for further optimization in non-treatment components such as preoperative diagnostics and basic nursing care.

Future development should establish standardized cost structures for open-heart surgery within DRG and Diagnosis-Intervention Packet (DIP) payment frameworks. These systems employ distinct but complementary approaches: DRG categorizes cases by diagnosis and procedure for case-based reimbursement, while DIP allocates payment points according to diagnosis-treatment combinations (19, 20). Together, these payment mechanisms promote cost optimization through standardized reimbursement benchmarks. This approach aims to define appropriate expenditure ranges for core cost categories including treatment fees and materials procurement, ultimately balancing technical service valuation with treatment affordability.

Material costs consistently constitute the predominant component within the cost structure of interventional procedures. During 2014–2024, these expenses fluctuated between 29.26% and 54.79% of total costs, demonstrating a gradual yet fluctuating decline. Median material costs peaked at ¥34,515.53 in 2015 before entering a steady downward trajectory from 2019 onward, ultimately reaching ¥8,764.98 by 2024. This substantial reduction has been instrumental in driving down overall expenditures for interventional procedures.

The observed cost containment aligns temporally with the volume-based procurement policy implemented under the 2019 Reform Plan for High-Value Medical Consumables, which effectively reduced market prices for essential devices including occlusion equipment and vascular stents. Nevertheless, persistent price differentials between domestic and imported alternatives continue to constrain further material cost optimization. Domestic medical devices not only provide superior cost-effectiveness but have also demonstrated clinical efficacy equivalent to imported products, as validated by authoritative guidelines such as the Common Congenital Heart Disease Percutaneous Interventional Surgery Guidelines (2021 Edition).

Despite these advantages, several barriers limit broader adoption of domestic devices, including persistent misconceptions among patients' families and preferential prescribing practices favoring imported alternatives among some clinicians. To address these challenges, a dual-strategy approach is recommended: first, implementing evidence-based communication initiatives that utilize clinical outcome data from regional treatment centers to objectively demonstrate the therapeutic equivalence and economic advantages of domestic devices; second, establishing standardized clinical pathways and performance metrics that explicitly recommend domestically produced devices for appropriate simple-lesion cases, while maintaining respect for informed patient choice within established regulatory parameters.

While minimally invasive approaches, particularly interventional procedures, provide distinct advantages in reduced trauma and cost containment, broader implementation of minimally invasive cardiac surgery faces several practical constraints. These include significant technical complexity, extended learning curves for surgical teams, and limited cost-effectiveness evidence for specialized equipment. The successful application of these techniques requires specialized surgical teams selecting appropriate patient candidates to ensure optimal minimally invasive outcomes (21). Therefore, advancing these capabilities necessitates dedicated technical training programs to enhance team proficiency. Promoting standardized application in suitable clinical cases will ultimately optimize both healthcare expenditures and clinical outcomes, thereby improving accessibility to minimally invasive therapeutic options for pediatric CHD patients in Xinjiang.

### 4.2 Analysis of demographic characteristics

Patients with CHD received treatment across all pediatric age groups, reflecting variations in diagnostic timing and surgical indications. The 2–5 years age group represented the largest proportion (36.04%), a pattern reflecting the combined influence of cardiovascular developmental characteristics, diagnostic and therapeutic advancements, and public health initiatives. This “physiological treatment window” offers relative cardiac structural stability, more definitive clinical signs, and enhanced surgical tolerance (22–25). Concurrently, the widespread availability of imaging and catheterization technologies enables accurate early diagnosis (26), while systematic screening through kindergarten entrance health examinations [implemented nationally in 2010 (27)] aligns the preschool age range (3–6 years) with this optimal treatment period, collectively explaining the concentration of cases in this age group.

A significant inverse relationship emerged between age and hospitalization costs, with children under 2 years incurring the highest median costs (¥61,657.91) and adolescents aged 12–14 years the lowest (¥45,120.19). This pattern reflects the clinical reality of immature organ function and elevated perioperative complication risks in younger patients, necessitating more intensive medical monitoring—consistent with the established principle of higher resource consumption at younger ages.

Notably, over half (52.80%) of the children lacked health insurance coverage at the time of treatment. Despite national policies enabling immediate post-birth insurance enrollment (28), awareness remains limited in Xinjiang's rural and pastoral regions, likely due to geographic dispersion and insufficient policy dissemination. Uninsured children faced significantly higher median hospitalization costs (¥53,872.79) than their insured counterparts (¥47,746.36), exacerbating out-of-pocket household expenses and potentially creating a detrimental cycle of treatment delays leading to compounded financial burdens and increased catastrophic health expenditure risk. Consequently, enhancing policy communication and expanding newborn insurance enrollment represent urgent priorities for addressing this challenge.

Atrial septal defect (44.47%) and ventricular septal defect (32.19%) emerged as the most common simple congenital heart diseases in our cohort, aligning with epidemiological patterns reported in domestic and international multicenter studies (29, 30). Regarding disease severity, 54.54% of patients (1,533 cases) presented with complex severe forms, demonstrating significantly higher median hospitalization costs (¥52,468.64) compared to simple CHD cases (¥50,121.31). Open-heart surgery represented the predominant treatment approach (64.35%), reflecting both its irreplaceable role in managing complex conditions such as tetralogy of Fallot (31) and the established technical expertise within Xinjiang's healthcare system (27). In comparison, interventional procedures provide a viable alternative for selected simple lesions, with significantly lower median costs (¥47,233.91) than open-heart surgery (¥54,573.63), suggesting that strategic promotion of interventional techniques represents a promising approach for cost containment. However, the application of interventional procedures requires careful patient selection based on individual anatomical considerations and clinical status (32). Hospitalization duration primarily ranged between 7 and 15 days in our study, exceeding the 5–7 day ideal standard recommended by the Clinical Pathway for Pediatric Congenital Heart Disease, with open-heart procedures particularly associated with extended stays. This discrepancy appears closely related to several factors, including delayed recovery in younger children, elevated postoperative complication risks, and inherent disease complexity.

### 4.3 Pathway analysis of hospitalization cost determinants and policy implications

#### 4.3.1 Age factor

Age demonstrated a significant negative indirect influence on hospitalization costs (path coefficient: β = −0.047), indicating that older pediatric patients generally incurred lower medical expenditures. This association can be attributed to several age-related physiological and clinical considerations. Older children typically present with more developed cardiovascular systems and a higher prevalence of simple CHD types, making them better candidates for interventional procedures. Furthermore, their more mature immune systems contribute to reduced complication rates and shorter recovery periods. These advantages collectively create a dual pathway for cost containment: increased eligibility for less invasive interventions and decreased need for extended medical supervision. Consequently, developing age-differentiated clinical protocols appears warranted, while healthcare payers might consider establishing age-stratified reimbursement standards within DRG/DIP payment models to better align with the distinct cost structures of different pediatric age groups.

#### 4.3.2 Disease type factors

Disease type demonstrated a significant positive indirect effect on hospitalization costs (path coefficient: β = 0.087). The underlying mechanism involves how anatomical variations across different CHD types directly determine treatment selection. Complex congenital anomalies frequently necessitate open-heart surgery, which—due to its invasive nature and extended recovery requirements—prolongs hospitalization duration, thereby increasing costs. Consequently, we recommend that clinical departments establish standardized treatment pathways based on disease classification, prioritizing interventional approaches for eligible simple lesions while enhancing patient education about interventional procedure advantages. Additionally, strengthening public health screening initiatives through improved prenatal screening and early neonatal diagnosis would facilitate “early detection, diagnosis, and treatment” (33), enabling intervention at less severe disease stages. Multidisciplinary collaboration to optimize perioperative management could further reduce hospitalization duration, while health insurance status policies should consider incorporating disease-type differentiation within DRG/DIP payment systems to incentivize cost-effective treatment selection.

#### 4.3.3 Disease severity factors

Disease severity demonstrated a significant positive indirect effect on hospitalization costs (path coefficient: β = 0.061). Patients with more severe conditions showed increased likelihood of undergoing open-heart surgical procedures. These invasive interventions, characterized by substantial tissue trauma and extended recovery requirements, typically resulted in prolonged hospitalization, consequently elevating medical expenditures. In accordance with the objectives of Diagnosis-Related Group (DRG) and Diagnosis-Intervention Packet (DIP) payment reforms (34), we recommend establishing stratified clinical pathways based on disease severity classification. These pathways should specify preferred treatment algorithms and standardized hospitalization durations for each severity tier. Furthermore, incorporating key performance indicators such as length of stay into the DRG/DIP payment framework would incentivize healthcare institutions to proactively optimize resource allocation and effectively control medical costs.

#### 4.3.4 Health insurance factors

Health insurance demonstrated a significant negative indirect effect on hospitalization costs through the pathway of hospitalization duration (path coefficient: β = −0.045). This relationship may originate from multiple mechanisms: insurance reimbursement policies, price regulation mechanisms, and clinical pathway standardization directly constrain medical expenditures (35). Simultaneously, the cost-sharing function of insurance coverage reduces financial barriers for families, preventing treatment delays and enabling earlier medical intervention at less severe disease stages, thereby shortening hospital stays.

However, potential selection bias requires careful consideration. Insured families may possess socioeconomic advantages that facilitate access to higher-quality medical resources, and their case composition might include a greater proportion of simple CHD types. These factors could interact with insurance policy effects to jointly influence hospitalization duration and costs. Although this observational study employed multivariate statistical methods to control for potential confounders, residual bias cannot be completely ruled out. Therefore, the independent effect of health insurance should be interpreted with appropriate caution. Future investigations should incorporate detailed socioeconomic variables, particularly household income data, to more precisely evaluate the net effect of insurance coverage on medical expenditures.

## 5 Conclusions

This study provides a systematic analysis of hospitalization cost characteristics and their underlying mechanisms, drawing upon retrospective data from 2,811 pediatric CHD patients treated at a tertiary hospital in Xinjiang between 2013 and 2024. The results revealed a distinct “rise-then-fall” pattern in median hospitalization costs, with sustained reduction observed after 2019. Open-heart surgery consistently incurred higher costs than interventional procedures, with significant differences in their respective cost structures. Path analysis identified hospitalization duration, disease severity, and treatment year as core direct determinants of costs, while age, disease type, and insurance status operated through the critical pathway of “treatment approach → length of stay” to exert significant indirect effects. Furthermore, calculated out-of-pocket expenditures substantially exceeded the catastrophic health expenditure threshold, indicating substantial economic pressure on affected families. Consequently, we recommend coordinated implementation of four key strategies: promoting interventional techniques, optimizing health insurance reimbursement mechanisms, establishing severity-stratified clinical pathways, and creating targeted financial assistance programs. This study has several limitations. First, its single-center retrospective design may affect the generalizability of the findings. Second, although costs were adjusted for inflation, the twelve-year study period encompasses inherent temporal variability in healthcare pricing, along with policy and technological shifts that are difficult to fully account for—factors that should be considered when interpreting long-term trends. Future multicenter prospective studies are needed to validate these results.

## Data Availability

The data analyzed in this study is subject to the following licenses/restrictions: due to data confidentiality, the data cannot be disclosed. Requests to access these datasets should be directed to Jiangabieke Lizha, 474536527@qq.com.
